# TIM-3 Genetic Variants Are Associated with Altered Clinical Outcome and Susceptibility to Gram-Positive Infections in Patients with Sepsis

**DOI:** 10.3390/ijms21218318

**Published:** 2020-11-06

**Authors:** Caspar Mewes, Tessa Alexander, Benedikt Büttner, José Hinz, Ayelet Alpert, Aron-F. Popov, Michael Ghadimi, Tim Beißbarth, Mladen Tzvetkov, Marian Grade, Michael Quintel, Ingo Bergmann, Ashham Mansur

**Affiliations:** 1 Department of Anesthesiology, University Medical Center, Georg August University, D-37075 Goettingen, Germany; tessa.alexander@med.uni-goettingen.de (T.A.); benedikt.buettner@med.uni-goettingen.de (B.B.); mquintel@med.uni-goettingen.de (M.Q.); ingo.bergmann@med.uni-goettingen.de (I.B.); 2 Department of Anesthesiology and Intensive Care Medicine, Klinikum Region Hannover, D-30459 Hannover, Germany; jose.hinz@krh.eu; 3 Department of Immunology, Rapport Faculty of Medicine, Technion-Israeli Institute of Technology, Haifa 31096, Israel; ayelethappy@gmail.com; 4 Department of Thoracic and Cardiovascular Surgery, University Medical Center, Eberhard Karls University, D-72076 Tuebingen, Germany; aronf.popov@gmail.com; 5 Department of General and Visceral Surgery, University Medical Center, Georg August University, D-37075 Goettingen, Germany; mghadim@uni-goettingen.de (M.G.); marian.grade@med.uni-goettingen.de (M.G.); 6 Institute of Medical Bioinformatics, University Medical Center, Georg August University, D-37077 Goettingen, Germany; tim.beissbarth@ams.med.uni-goettingen.de; 7 Department of Pharmacology, University Medical Center, Ernst-Moritz-Arndt-University, D-17487 Greifswald, Germany; mladen.tzvetkov@uni-greifswald.de; 8 Department of Anesthesiology, Asklepios Hospitals Schildautal, D-38723 Seesen, Germany

**Keywords:** TIM-3, sepsis, single nucleotide polymorphism (SNP), mortality, predictor, Gram-positive infections

## Abstract

**Background**: Previous studies have reported the fundamental role of immunoregulatory proteins in the clinical phenotype and outcome of sepsis. This study investigated two functional single nucleotide polymorphisms (SNPs) of T cell immunoglobulin and mucin domain-containing protein 3 (TIM-3), which has a negative stimulatory function in the T cell immune response. **Methods**: Patients with sepsis (*n* = 712) were prospectively enrolled from three intensive care units (ICUs) at the University Medical Center Goettingen since 2012. All patients were genotyped for the TIM-3 SNPs rs1036199 and rs10515746. The primary outcome was 28-day mortality. Disease severity and microbiological findings were secondary endpoints. **Results**: Kaplan–Meier survival analysis demonstrated a significantly lower 28-day mortality for TIM-3 rs1036199 AA homozygous patients compared to C-allele carriers (18% vs. 27%, *p* = 0.0099) and TIM-3 rs10515746 CC homozygous patients compared to A-allele carriers (18% vs. 26%, *p* = 0.0202). The TIM-3 rs1036199 AA genotype and rs10515746 CC genotype remained significant predictors for 28-day mortality in the multivariate Cox regression analysis after adjustment for relevant confounders (adjusted hazard ratios: 0.67 and 0.70). Additionally, patients carrying the rs1036199 AA genotype presented more Gram-positive and *Staphylococcus epidermidis* infections, and rs10515746 CC homozygotes presented more *Staphylococcus epidermidis* infections. **Conclusion**: The studied TIM-3 genetic variants are associated with altered 28-day mortality and susceptibility to Gram-positive infections in sepsis.

## 1. Introduction

Sepsis, an inadequate immune response to infection, can be assumed to be a fundamental health care issue with a global reach. According to the World Health Organization (WHO), there are approximately 30 million sepsis episodes and, consequently, 6 million deaths per year [1,2,3]. Despite strong efforts to understand the pathogenesis and improve supportive treatment, sepsis still leads to markedly high in-hospital mortality as well as high rates of concomitant comorbidities and rehospitalizations [3,4,5,6,7]. The worldwide increasing incidence challenges intensive care units (ICUs) every day, thus leading to a social and economic burden and illustrating the urgent need for transition [3,6,8].

There is accumulating evidence for negative costimulatory immunoregulatory checkpoint proteins and their downregulation of an overwhelming T cell immune reaction significantly affecting the immunopathology of sepsis [9]. In particular, genetic variants in key genes of the host immune response, including programmed cell death protein 1 (PD-1) and cytotoxic T-lymphocyte associated protein 4 (CTLA-4) inter alia, have been studied in patients experiencing sepsis [10,11,12]. We suggest that a better categorization of patients with sepsis according to host genetic components could lead to the development of individualized therapeutic strategies, improved clinical outcomes, and enhanced patient prognosis [13].

TIM-3, T cell immunoglobulin and mucin domain-containing protein 3, is a type I transmembrane protein and belongs to the TIM family of immunoregulatory proteins [14]. Expressed on CD4-positive T cells (CD4+) and on CD8-positive cytotoxic T cells (CD8+) as well as on natural killer cells (NKs), regulatory T cells (TRegs), myeloid cells, mast cells, and dendritic cells (DCs), TIM-3 is involved in both the innate and adaptive host immune responses [15,16,17]. Depending on its ligand, the expressing cell type, and the host immune status, TIM-3 affects the immune response with either inhibitory or costimulatory function [17]. Because of its crucial role in the regulation of the host immune response, TIM-3 has previously been correlated with the pathogenesis of many clinical diseases, such as liver diseases, hyperreactive airway diseases such as asthma, and several autoimmune diseases such as rheumatoid arthritis or type 1 diabetes [17,18,19,20,21,22,23].

The TIM-3 pathway is suggested to play an important role in maintaining immune homeostasis in sepsis [24]. There is evidence that the overexpression of TIM-3 suppresses Toll-like-receptor (TLR)-mediated proinflammatory cytokine production and reduces macrophage responsiveness [14,24]. Furthermore, the binding of TIM-3 to Galectin-9 (Gal-9), one of the most studied TIM-3 ligands, is reported to induce apoptosis in CD4+ T cells as well as CD8+ cytotoxic T cells [25,26,27].

However, intentions of blocking the TIM-3 pathway or TIM-3 together with other coinhibitory proteins, such as PD-1, in patients with sepsis showed inconsistent results [28,29]. Clinical trials so far cannot recommend a specific treatment tailored to the patient needs in sepsis [30,31].

In humans, single nucleotide polymorphisms (SNPs) in the coding and noncoding regions of the TIM-3 gene, also known by the gene name HAVCR2 (hepatitis A virus cellular receptor 2), have been linked to several allergic and autoimmune diseases [21,32,33]. Two important SNPs in the HAVCR2 region located on chromosome 5 were examined in the present study. Both the functional TIM-3 rs1036199 SNP, which encodes a missense mutation, and the TIM-3 rs10515746 SNP, an upstream variant in the promoter region, have previously been associated with increased susceptibility to autoimmune diseases such as rheumatoid arthritis and multiple sclerosis [21,32,33,34,35].

Considering the inhibitory function of TIM-3 in the T cell immune response and the important role of the TIM-3 rs1036199 and rs10515746 SNPs in immune regulation and susceptibility to autoimmune diseases, we hypothesized that these genetic variants may also affect the immunopathology, disease progression, and clinical outcome of patients with sepsis. The present study is the first to explore the association of the TIM-3 rs1036199 and rs10515746 genotypes with altered clinical outcomes in sepsis. Twenty-eight-day mortality served as the primary outcome parameter, while disease severity and microbiological findings presented the secondary endpoints of this investigation.

## 2. Materials and Methods

### 2.1. Patient Collective

This study involved 712 Caucasian patients with clinically defined sepsis. They were prospectively enrolled from three surgical ICUs through the GENOSEP database of the Department of Anesthesiology at the University Medical Center, Goettingen, Germany, as previously described [36,37]. Since 2012, patients with sepsis have been recruited by daily screening of the three ICUs according to the current sepsis definitions and guidelines [7,38].

The following exclusion criteria were applied: (I) less than 18 years of age; (II) pregnancy and/or breastfeeding; (III) therapy with immunosuppressive drugs, such as cyclosporine A, glucocorticoids or methotrexate, and/or chemotherapy within six months pre-enrollment; (IV) myocardial infarction within six weeks before recruitment; (V) chronic heart failure classified as New York Heart Association (NYHA) stage IV; (VI) human immunodeficiency virus (HIV) infection and/or hepatitis B/C infection; (VII) end-stage incurable disease; (VIII) a persistent vegetative state (apallic syndrome); (IX) a “Do Not Treat (DNT)” or “Do Not Resuscitate” (DNR) order; (X) consistent participation in interventional studies and (XI) family members of a study-site employee.

The present investigation was approved by the institutional ethics committee of the University of Goettingen in Goettingen, Germany, under the ethical project identification code 15/1/12, and respected the ethical provisions of the Declaration of Helsinki. The study was performed in accordance with approved guidelines. Written informed consent was obtained either from the patient or their legal representative.

### 2.2. Data Collection

A standardized clinical report form (CRF) was used to follow-up patients for a period of 28 days after sepsis onset, and the anonymized data were collected in the GENOSEP database. All study participants were followed up for 28 days unless previously dismissed from the study or deceased. In order to obtain the primary outcome parameter, patient survival was confirmed after 28 days by individual telephone calls or inquiries at the local registration office.

Relevant baseline characteristics, such as sex, age, premedication, preexisting comorbidities, recent surgical history, and initial Sequential Organ Failure Assessment (SOFA) and Acute Physiology and Chronic Health Evaluation (APACHE II) scores, were recorded. To measure organ failure, disease severity was calculated continuously on the basis of the daily SOFA scores, organ-specific SOFA subscores, and relevant organ support parameters such as ventilated, vasopressed, or dialyzed days. Additional inflammatory variables, e.g., procalcitonin, C-reactive protein (CRP), or temperature, were also recorded. All microbiological findings of the admitted study participants were recorded over the observation period and categorized into Gram-positive, Gram-negative, viral, and fungal. Only infections with clinical evidence for an infection were considered, while assumed false positive cultures due to contamination or colonization were dismissed. Microbiological analyses included probes from tracheal secretions, nasopharyngeal swabs, blood cultures, abdominal or intrasurgical swabs, feces, and the urinary system. Predominant specific pathogens, such as *Staphylococcus aureus* or *Candida albicans,* were specifically examined in the performed microbiological analysis. All patient data were generated from the electronic patient record system (IntelliSpace Critical Care and Anesthesia (ICCA), Phillips Healthcare, Andover, MA, USA).

### 2.3. Genotyping

All experimental protocols for DNA extraction and genotyping were performed in the laboratories and under the supervision of the Department of Clinical Pharmacology at the University Medical Center Goettingen. Blood was drawn from all study participants within 72 h after sepsis onset. Genomic DNA was extracted either from 200 µL of ethylenediaminetetraacetic acid (EDTA) blood using a QIAmp^®^ DNA Blood Kit in QIAcube^®^, from 350 µL of EDTA blood using an EZ1^®^ DNA Blood Kit in BioRobot EZ1^®^ or from peripheral blood mononuclear cells (PBMCs) using an AllPrep DNA Mini Kit according to the manufacturer’s instructions (all from Qiagen, Hilden, Germany). For the purpose of quantity and quality controls, the extracted DNA was tested by spectrophotometric measurement.

Genotyping of TIM-3 rs1036199 and rs10515746 was performed through TaqMan polymerase chain reaction (PCR) using the appropriate predesigned TaqMan^®^ SNP Genotyping Assays C_2082038_1_and C_2082054_20 and a 7900HT Fast-Real-Time PCR System (Life Technologies, Darmstadt, Germany) as well as 7900HT Fast-Real-Time PCR System software (SDS v2.4.1 for Windows 7, Applied Biosystems, Foster City, CA, USA). A minimum of 20% of the samples were genotyped in duplicate to verify the primary measurement results and increase the reliability.

### 2.4. Statistical Analysis

STATISTICA 13 software (version 13.0, StatSoft, Tulsa, OK, USA) was used for all statistical analyses. For the presented data, a *p* value < 0.05 was considered statistically significant.

The Mann–Whitney U test was used for all continuous variables, whereas Pearson’s chi-square test or a two-sided Fisher’s exact test was used for discrete variables in the analyses of patient baseline characteristics, disease severity, and microbiological findings. Continuous variables are listed as the mean ± standard deviation, and the results of discrete variables are presented as absolute numbers or percentages.

The log-rank test was used for the Kaplan–Meier survival analyses. Accordance with Hardy–Weinberg equilibrium (HWE) was tested by the chi-square test, and linkage disequilibrium (LD) analysis was performed using Haploview^®^ software (Version 4.2, Broad Institute of MIT and Harvard, Cambridge, MA, USA) with regard to a representative HapMap reference population. To eliminate the effect of potential confounders such as age, sex, body mass index (BMI), and SOFA and APACHE II scores as well as significant findings from the patient baseline characteristics analysis on the outcome, multivariate Cox regression analyses were performed, and adjusted hazard ratios were estimated.

### 2.5. Data Availability

The datasets generated and/or analyzed during the current study are available from the corresponding author on reasonable request.

## 3. Results

### 3.1. Allele Distribution and Linkage Disequilibrium Analysis

All 712 study participants were successfully genotyped for the TIM-3 SNPs rs1036199 and rs10515746. The observed allele distributions were 471:216:25 (AA:AC:CC) for TIM-3 rs1036199 and 465:220:27 (CC:AC:AA) for TIM-3 rs10515746. Hence, minor allele frequencies (MAFs) of 0.187 for TIM-3 rs1036199 and 0.195 for TIM-3 rs10515746 were calculated, which nearly equaled those of the 1000 Genomes reference population (0.182 for TIM-3 rs1036199 [34] and 0.194 for TIM-3 rs10515746 [35], respectively). The observed allele frequencies were in accordance with Hardy–Weinberg equilibrium (χ^2^ test *p* = 0.9693 for TIM-3 rs1036199 and χ^2^ test *p* = 0.8775 for TIM-3 rs10515746).

Carriers of the TIM-3 rs1036199 AC genotype were pooled with carriers of the CC genotype (combined *n* = 241) and compared to AA homozygous patients (*n* = 471) in all of the following analyses. Likewise, the TIM-3 rs10515746 A-allele carriers (AA and AC genotypes) were pooled (combined *n* = 247) and compared to the CC homozygotes (*n* = 465).

LD analysis was performed for the studied SNPs and revealed an LD coefficient (D′) of 1.0 (0.98–1.0) and a squared correlation coefficient (r^2^) of 0.925. Consequently, the TIM-3 rs1036199 A-allele is correlated with the rs10515746 C-allele.

### 3.2. Baseline Characteristics

The examined cohort of 712 prospectively enrolled adult patients with clinically defined sepsis showed no significant differences in baseline characteristics regarding age, sex, disease severity on sepsis onset, preexisting conditions, recent surgical history, or site of infection between the compared TIM-3 genetic variants (Table 1 and Table 2). However, the use of bronchodilators as preexisting medication on sepsis onset was significantly higher in TIM-3 rs1036199 AA homozygous patients compared to C-allele carriers (13% vs. 6%; *p* = 0.0052; Table 1) and in TIM-3 rs10515746 CC homozygous patients compared to A-allele carriers (12% vs. 6%; *p* = 0.0155; Table 2).

The study participants were on average 63 ± 15 years old, and 65% of them were men. With a mean BMI of 28 ± 7, the cohort was in the upper range of normal weight. The average SOFA and APACHE II scores on sepsis onset measured 10 ± 4 and 22 ± 7, indicating the need for intensive care support. At baseline, 86% of the patients were mechanically ventilated, 70% received vasopressors, and 10% were in need of renal replacement therapy. At 54%, arterial hypertension was the most common comorbidity, followed by COPD (15%) and history of cancer (14%). Approximately half of the patients were undergoing emergency surgery prior to sepsis onset, and in 63%, the lung was the major site of infection.

### 3.3. Kaplan–Meier Survival Analysis

**Figure 1:**
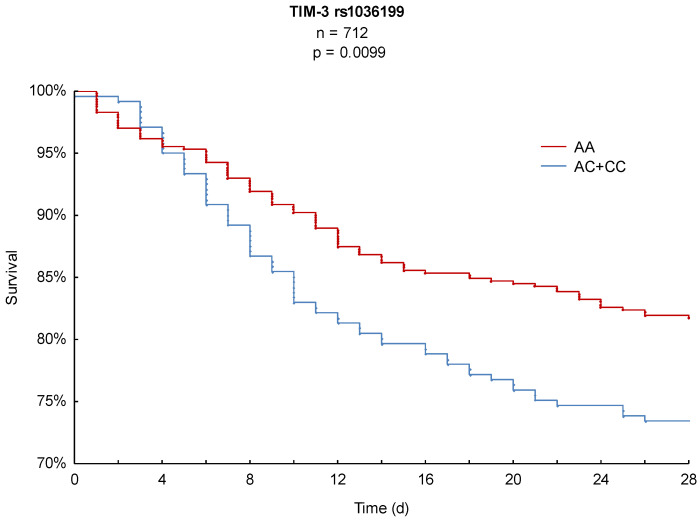
Kaplan–Meier 28-day survival analysis with respect to TIM-3 rs1036199.

**Figure 2:**
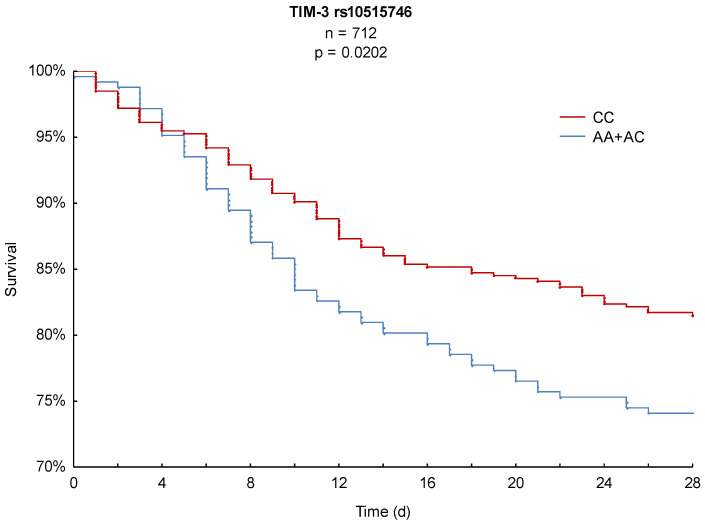
Kaplan–Meier 28-day survival analysis with respect to TIM-3 rs10515746.

In order to achieve the primary outcome parameter, 28-day Kaplan–Meier survival analysis was performed and revealed significant differences in mortality for both SNPs. As illustrated in Figure 1, AA homozygous patients of TIM-3 rs1036199 presented a significantly lower 28-day mortality of 18% compared to C-allele carriers with 27% (*p* = 0.0099). Likewise, TIM-3 rs10515746 CC homozygous patients showed a significantly decreased hazard of death over the 28-day observation period of 18% in comparison to A-allele carriers, with a 28-day mortality of 26% (*p* = 0.0202, Figure 2).

### 3.4. Disease Severity

The conducted disease severity analyses were structured into the main categories of general sepsis severity, inflammatory values, and different organ-specific parameters, including respiration, coagulation, liver values, cardiovascular system, central nervous system, and renal values (Table 3 and Table 4). No significant differences in disease severity or organ dysfunction were observed between the studied TIM-3 rs1036199 and TIM-3 rs10515746 genotypes.

### 3.5. Microbiological Analysis

**Figure 3:**
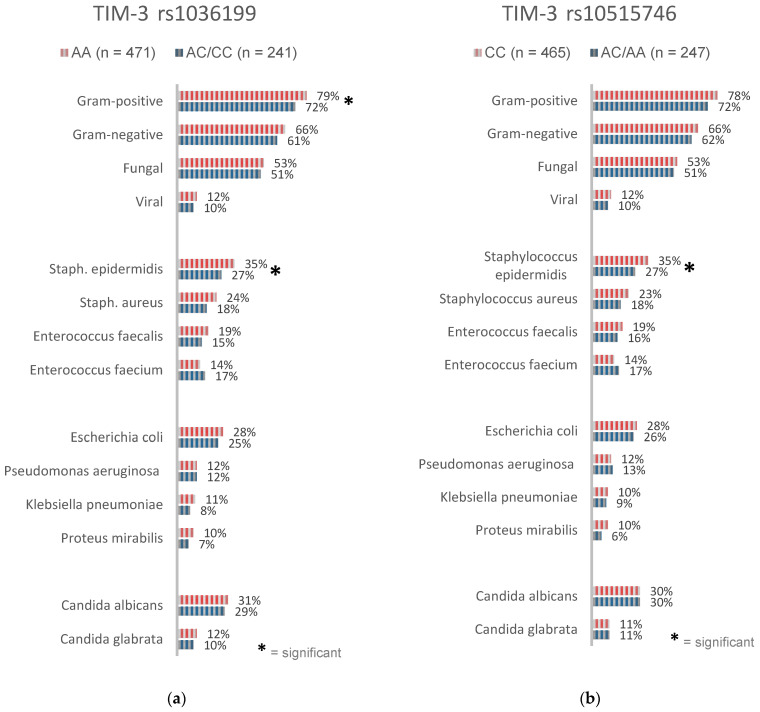
Frequencies of the type of infection and specific pathogens according to (**a**) TIM-3 rs1036199 and (**b**) TIM-3 rs10515746 genotypes.

The performed analyses of microbiological findings examined the incidences of several specific pathogens associated with nosocomial infections as well as the general type of infection (Gram-positive, Gram-negative, viral, fungal; Figure 3, Supplementary Material). For the TIM-3 rs1036199 SNP, a significantly higher rate of Gram-positive infections was observed in AA homozygous patients (79%) compared to C-allele carriers (72%; *p* = 0.0445; Supplementary Material). Furthermore, carriers of the AA genotype at this position presented a higher incidence of *Staphylococcus epidermidis* infections (35%) in comparison to C-allele carriers (27%; *p* = 0.0295; Supplementary Material).

Likewise, carriers of the CC genotype at the TIM-3 rs10515746 position showed a significantly higher rate of infections with *Staphylococcus epidermidis* than the A-allele carriers (35% vs. 27%; *p* = 0.0313; Supplementary Material). The analyses of further pathogens could not demonstrate additional significant differences between the groups.

Both the TIM-3 rs1036199 AA genotype and the TIM-3 rs10515746 CC genotype presented higher frequencies of almost all analyzed pathogens and the type of infection compared to other genotypes at these positions (Figure 3). However, the majority of the observed differences in pathogen frequencies did not reach statistical significance.

### 3.6. Multivariate Cox Regression Analysis

The impact of potential confounders and previously observed significance in the patient baseline characteristics on the 28-day mortality was considered in the multivariate Cox regression analyses of this study. Age, sex, BMI, SOFA, and APACHE II score on sepsis onset were included as potential confounders in these analyses. In addition, the use of bronchodilators as preexisting medication was considered in accordance with previous findings in Table 1 and Table 2.

The conducted multivariate model revealed that age, BMI, SOFA, APACHE II, and both the TIM-3 rs1036199 AA genotype as well as the TIM-3 rs10515746 CC genotype significantly affected the 28-day mortality. While increased age, male sex, and higher SOFA and APACHE scores at sepsis onset negatively affected 28-day mortality, BMI was identified to have a positive effect.

Furthermore, carrying the TIM-3 rs1036199 AA genotype (adjusted hazard ratio: 0.6714; 95% CI: 0.4825–0.9341; *p* = 0.0180; Table 5) and/or the TIM-3 rs10515746 CC genotype (adjusted hazard ratio: 0.6981; 95% CI: 0.5021–0.9706; *p* = 0.0326; Table 6) remained as having a significantly positive effect after adjustment for confounders and may therefore serve as significant predictors of 28-day survival.

## 4. Discussion

As the main finding, the present study is the first to reveal the association of the TIM-3 genetic variants TIM-3 rs1036199 AA and the TIM-3 rs10515746 CC with improved 28-day survival in sepsis. The associations remained significant positive predictors for 28-day survival of sepsis after adjustment for relevant confounders in the multivariate Cox regression model.

Despite strong efforts in understanding the immunopathology of sepsis, this life-threatening disease has still not been completely explored, and the mortality rate remains unacceptably high [1,7]. In order to achieve improvements towards better patient categorization, improved diagnostics, appropriate treatment, and identification of high-risk patients, there is an urgent need for identification of disease-associated host genetic components [13]. Thus, along with several other host genetic variants, TIM-3, a transmembrane protein with dampening effects on inflammation in sepsis, has become the focus of our interest. As mentioned earlier, intentions to affect the outcome of sepsis by blocking TIM-3 itself or blocking TIM-3 along with other coinhibitory proteins showed inconsistent results in previous studies [28,29].

The two functional TIM-3 SNPs rs1036199 and rs10515746 have been investigated in association with autoimmune diseases, but the influence of these specific SNPs on the progression and outcome of sepsis remains unclear [32]. As the primary outcome, this study suggests that genetic variations at the TIM-3 rs1036199 and rs10515746 positions are significantly associated with 28-day mortality in patients with sepsis. Both the TIM-3 rs1036199 AA and the TIM-3 rs10515746 CC genotype are associated with a significantly improved 28-day survival in contrast to the C-allele carriers of TIM-3 rs1036199 (18% vs. 27%; *p* = 0.0099) and A-allele carriers of TIM-3 rs10515746 (18% vs. 26%; *p* = 0.0202), respectively. These findings remained significant after including several potential confounders in the multivariate Cox regression model, yielding an estimated adjusted hazard ratio of 0.6714 (95% CI: 0.4825–0.9341; *p* = 0.0180) for the TIM-3 rs1036199 AA genotype and 0.6981 (95% CI: 0.5021–0.9706; *p* = 0.032) for the TIM-3 rs10515746 CC genotype, indicating the independent prognostic value of these genetic variants.

The TIM-3 rs1036199 SNP on exon 3 of the TIM-3 gene leads to an amino acid substitution from arginine to leucine and thereby causes alterations in the structure and function of the TIM-3 protein [33,34]. Furthermore, the TIM-3 rs10515746 SNP is located in the promotor region of the TIM-3 gene and is highly linked with the TIM-3 rs1036199 SNP [33,35]. The observed allele changes in the two SNPs could therefore result in altered protein function as well as altered regulation of TIM-3 expression [21,23]. As a possible explanation for the observed findings, the genetic variants with favorable outcomes could have led to these alterations in TIM-3 protein function and expression and may have a significant impact on immune homeostasis in sepsis. Reduced expression of TIM-3 or attenuated protein function may have been responsible for increased TLR-mediated proinflammatory cytokine production and macrophage responsiveness as well as reduced Gal-9-induced apoptosis in CD4+ and CD8+ T-cells [26,39,40,41]. The strengthened T-cell-mediated and macrophage immune reaction may have beneficial effects on the course of disease and outcome in sepsis but also predisposes for the development of autoimmune diseases by enhanced T effector cell function [32]. Our findings are consistent with previous studies that reported a correlation between the downregulation or blockade of TIM-3 and the severity of sepsis [24]. However, the underlying biological and pathophysiological mechanisms behind the observed findings remain unclear and need to be further investigated in future studies.

These explanatory approaches are further supported by the results of the performed analysis of patient baseline characteristics. Here, our investigations revealed a significantly increased need for bronchodilators on sepsis onset for the TIM-3 rs1036199 AA genotype analogous to the TIM-3 rs10515746 CC genotype. Although we could not observe significant differences in preexisting comorbidities such as COPD or bronchial asthma, these findings are consistent and further support previously detected correlations between inherited variants of TIM genes and airway hyperreactivity [18]. Furthermore, it supports our hypothesis that TIM-3 genetic variants with favorable outcomes (TIM-3 rs1036199 AA and TIM-3 rs10515746 CC) might have a strengthened T-cell-mediated immune reaction, which is beneficial in the clinical course of sepsis but at the same time leads to a higher susceptibility to autoimmune diseases or chronic inflammatory diseases such as COPD and bronchial asthma [42].

Moreover, the microbiological analysis findings, e.g., the type of infection and several pathogens, revealed a significantly higher rate of infections with Gram-positive pathogens in general and *Staphylococcus epidermidis* specifically in patients carrying the TIM-3 rs1036199 AA genotype. Additionally, our study yielded an increased frequency of *Staphylococcus epidermidis* infections in patients carrying the TIM-3 rs10515746 CC genotype. A trend towards a higher susceptibility to other Gram-positive, Gram-negative, or fungal infections was also observed but did, however, lack statistical significance. We suspect that an attenuated function and/or altered expression of TIM-3 could be responsible for an unbalanced homeostatic immune regulation and inflammatory response in these genetic variants. Higher frequencies of clinically confirmed infections may potentially be due to an increased TLR-mediated response, which was previously shown to be negatively regulated by TIM-3 [43,44]. However, the underlying mechanisms of the revealed associations remain largely unclear.

Besides the significant findings for 28-day mortality and susceptibility to certain infections, our study was not able to reveal associations between the investigated TIM-3 SNPs and the disease severity of sepsis, as measured by the organ-specific SOFA subscore, clinical parameters, and organ support values. The applied standard clinical assessment parameters may not have been appropriate to adequately represent the patients’ disease severity or organ dysfunction.

As with many studies of this nature, the present study certainly has some limitations. It was conducted as a single-center study and thus requires validation in other cohorts from different centers, most likely also involving other ethnicities. On the other hand, the prospective study design and relatively large and homogenous cohort of septic patients as well as the large number of recorded and analyzed parameters are strengths of this study. Another limitation of the present study is that it only involved patients with sepsis from the surgical ICU and did not involve patients from medical or neurological ICUs.

In summary, this study is the first to reveal the impact of the TIM-3 rs1036199 and rs10515746 SNPs on 28-day mortality in sepsis and the frequencies of Gram-positive infections. The identified genetic variants may be helpful as prognostic variables and for the identification of high-risk patients. Furthermore, understanding the underlying mechanisms and the function of TIM-3 in the course of disease in sepsis may lead to improved diagnostics and/or innovative therapeutic approaches (e.g., personalized according to the TIM-3 genotype) in sepsis.

## Figures and Tables

**Table 1:** Patient baseline characteristics with regard to the TIM-3 rs1036199 genotypes.

Characteristics	All (*n* = 712)	AC + CC (*n* = 241)	AA (*n* = 471)	*p*-Value
**Basic Conditions**				
Age [years]	63 ± 15	63 ± 15	63 ± 15	0.9015
Male sex [%]	65	68	64	0.3231
Body mass index (BMI) [kg/m^2^]	28 ± 7	28 ± 8	28 ± 6	0.7304
**Severity on Sepsis Onset (Day 1)**				
Sequential Organ Failure Assessment (SOFA)	10 ± 4	10 ± 4	10 ± 4	0.9595
Acute Physiology And Chronic Health Evaluation (APACHE II)	22 ± 7	21 ± 7	22 ± 7	0.1826
Procalcitonin [ng/dl]	10 ± 29	13 ± 34	9 ± 25	0.5192
Use of vasopressor [%]	70	71	70	0.6323
Mechanical ventilation [%]	86	85	87	0.3360
Renal replacement therapy [%]	10	12	8	0.0935
**Comorbidities [%]**				
Arterial hypertension [%]	54	54	53	0.7872
Chronic obstructive pulmonary disease (COPD) [%]	15	13	16	0.2199
Bronchial asthma [%]	3	2	3	0.2911
Renal dysfunction [%]	10	12	9	0.2626
Non-insulin-dependent diabetes mellitus (NIDDM) [%]	9	8	9	0.7818
Insulin-dependent diabetes mellitus (IDDM) [%]	10	10	10	0.8687
Chronic liver disease [%]	6	6	6	0.9669
History of myocardial infarction [%]	5	5	6	0.9443
History of stroke [%]	6	5	6	0.3825
History of cancer [%]	14	16	13	0.2670
**Medication on Sepsis Onset [%]**				
Statins [%]	23	24	23	0.5984
Beta-blocker [%]	37	38	36	0.5859
ACE inhibitor [%]	29	31	28	0.4926
Bronchodilator [%]	10	6	13	**0.0052**
Diuretics [%]	33	34	33	0.7649
Anticoagulation during the last 6 months [%]	25	28	24	0.1974
**Recent Surgical History [%]**				
Elective surgery [%]	27	24	29	
Emergency surgery [%]	52	54	51	0.3330
No surgery [%]	21	22	20	
**Site of Infection [%]**				
Lung [%]	63	61	64	
Abdomen [%]	19	22	17	
Bone or soft tissue [%]	3	2	4	
Surgical wound [%]	2	2	1	0.4211
Urogenital [%]	2	2	3	
Primary bacteremia [%]	6	6	6	
Other [%]	5	5	5	

*p*-values calculated using Mann–Whitney U test, Pearson’s chi-square test, or two-sided Fisher’s exact test.

**Table 2:** Patient baseline characteristics with regard to the TIM-3 rs10515746 genotypes.

Characteristics	All (*n* = 712)	AA + AC (*n* = 247)	CC (*n* = 465)	*p*-Value
**Basic Conditions**				
Age [years]	63 ± 15	63 ± 15	63 ± 15	0.9536
Male sex [%]	65	68	64	0.2451
Body mass index (BMI) [kg/m^2^]	28 ± 7	28 ± 8	28 ± 6	0.7976
**Severity on Sepsis Onset (Day 1)**				
Sequential Organ Failure Assessment (SOFA)	10 ± 4	10 ± 4	10 ± 4	0.9343
Acute Physiology And Chronic Health Evaluation (APACHE II)	22 ± 7	21 ± 7	22 ± 7	0.1738
Procalcitonin [ng/dl]	10 ± 29	12 ± 34	9 ± 25	0.7732
Use of vasopressor [%]	70	70	69	0.7857
Mechanical ventilation [%]	86	85	87	0.3181
Renal replacement therapy [%]	10	12	9	0.2123
**Comorbidities [%]**				
Arterial hypertension [%]	54	53	54	0.6908
Chronic obstructive pulmonary disease (COPD) [%]	15	13	16	0.3269
Bronchial asthma [%]	3	2	3	0.5325
Renal dysfunction [%]	10	12	9	0.3401
Non-insulin-dependent diabetes mellitus (NIDDM) [%]	9	8	9	0.6736
Insulin-dependent diabetes mellitus (IDDM) [%]	10	10	10	0.9953
Chronic liver disease [%]	6	6	6	0.7929
History of myocardial infarction [%]	5	5	6	0.8546
History of stroke [%]	6	5	6	0.5211
History of cancer [%]	14	17	12	0.1095
**Medication on Sepsis Onset [%]**				
Statins [%]	23	24	23	0.6532
Beta-blocker [%]	37	38	36	0.7305
ACE inhibitor [%]	29	32	28	0.2832
Bronchodilator [%]	10	6	12	**0.0155**
Diuretics [%]	33	34	33	0.8960
Anticoagulation during the last 6 months [%]	25	28	24	0.3142
**Recent Surgical History [%]**				
Elective surgery [%]	27	25	29	
Emergency surgery [%]	52	54	51	0.5589
No surgery [%]	21	21	21	
**Site of Infection [%]**				
Lung [%]	63	62	64	
Abdomen [%]	19	22	17	
Bone or soft tissue [%]	3	2	4	
Surgical wound [%]	2	2	2	0.2890
Urogenital [%]	2	2	3	
Primary bacteremia [%]	6	6	6	
Other [%]	5	5	5	

*p*-values calculated using Mann–Whitney U test, Pearson’s chi-square test, or two-sided Fisher’s exact test.

**Table 3:** Disease severity regarding the TIM-3 rs1036199 genotypes.

Characteristics	All (*n* = 712)	AC + CC (*n* = 241)	AA (*n* = 471)	*p*-Value
**Sepsis Severity**				
Sequential Organ Failure Assessment (SOFA)	7.2 ± 3.7	7.3 ± 3.8	7.2 ± 3.6	0.9064
Patients in septic shock [%]	50	53	49	0.3291
Days in septic shock	2 ± 3	2 ± 2	2 ± 3	0.5047
**Inflammatory Values**				
Leukocytes [1000/µL]	13.2 ± 5.0	13.7 ± 5.2	13.0 ± 4.9	0.0619
C-reactive Protein [mg/L]	150.6 ± 85.9	148.0 ± 81.3	152.1 ± 88.5	0.7630
Procalcitonin [ng/dL]	4.4 ± 10.4	4.9 ± 10.4	4.1 ± 10.4	0.1922
Fever [%]	88	88	87	0.7188
**Respiratory Values**				
SOFA respiratory subscore	2.0 ± 0.8	1.9 ± 0.8	2.0 ± 0.8	0.2940
Patients with mechanical ventilation [%]	94	95	93	0.5335
Ventilation days/observation days [%]	68 ± 32	66 ± 32	69 ± 32	0.3608
**Coagulation**				
SOFA coagulation subscore	0.4 ± 0.6	0.4 ± 0.7	0.4 ± 0.6	0.7182
Thrombocytes [1000/µL]	292 ± 151	292 ± 150	292 ± 151	0.6663
**Liver Values**				
SOFA hepatic subscore	0.4 ± 0.7	0.4 ± 0.7	0.4 ± 0.7	0.2591
Bilirubin [mg/dL]	1.2 ± 2.1	1.2 ± 1.9	1.3 ± 2.2	0.7229
AST (GOT) [IU/L]	179 ± 599	190 ± 574	174 ± 612	0.8124
ALT (GPT) [IU/L]	97 ± 195	98 ± 208	97 ± 187	0.4376
**Cardiovascular Values**				
SOFA cardiovascular subscore	1.6 ± 1.0	1.6 ± 1.1	1.6 ± 1.0	0.9143
Patients with vasopressor treatment [%]	81	81	81	0.8456
Vasopressor days/observation days [%]	37 ± 32	37 ± 33	36 ± 31	0.8398
**Central Nervous System**				
SOFA central nervous system	2.1 ± 1.1	2.0 ± 1.1	2.1 ± 1.1	0.1701
Glasgow Coma Scale (GCS)	9.8 ± 3.2	10.0 ± 3.3	9.7 ± 3.2	0.2311
**Renal Values**				
SOFA renal subscore	0.8 ± 1.2	0.9 ± 1.2	0.8 ± 1.2	0.2780
Creatinine [mg/dL]	1.2 ± 0.9	1.3 ± 0.9	1.2 ± 0.9	0.2977
Urine output [mL/d]	2906 ± 1345	2915 ± 1420	2901 ± 1307	0.7551
Urine output [mL/kg/d]	1.5 ± 0.8	1.5 ± 0.8	1.5 ± 0.8	0.9251
Patients with renal replacement therapy [%]	22	23	22	0.7266
Dialysis days/observation days [%]	11 ± 25	12 ± 26	10 ± 24	0.7023

*p*-values calculated using Mann–Whitney U test, Pearson’s chi-square test, or two-sided Fisher’s exact test.

**Table 4:** Disease severity regarding the TIM-3 rs10515746 genotypes.

Characteristics	All (*n* = 712)	AA + AC (*n* = 247)	CC (*n* = 465)	*p*-Value
**Sepsis Severity**				
Sequential Organ Failure Assessment (SOFA)	7.2 ± 3.7	7.2 ± 3.7	7.3 ± 3.7	0.7846
Patients in Septic Shock [%]	50	53	49	0.3326
Days in Septic Shock	2 ± 3	2 ± 2	2 ± 3	0.5087
**Inflammatory Values**				
Leukocytes [1000/µL]	13.2 ± 5.0	13.7 ± 5.2	13.0 ± 4.9	0.0550
C-reactive Protein [mg/L]	150.6 ± 85.9	149.5 ± 82.1	151.2 ± 88.2	0.9759
Procalcitonin [ng/dL]	4.4 ± 10.4	4.8 ± 10.3	4.2 ± 10.5	0.2529
Fever [%]	88	88	88	0.7687
**Respiratory Values**				
SOFA respiratory subscore	2.0 ± 0.8	1.9 ± 0.8	2.0 ± 0.8	0.3020
Patients with mechanical ventilation [%]	94	94	94	0.6794
Ventilation days/observation days [%]	68 ± 32	66 ± 32	69 ± 31	0.3060
**Coagulation**				
SOFA coagulation subscore	0.4 ± 0.6	0.4 ± 0.7	0.4 ± 0.6	0.5976
Thrombocytes [1000/µL]	292 ± 151	296 ± 155	290 ± 149	0.4882
**Liver Values**				
SOFA hepatic subscore	0.4 ± 0.7	0.4 ± 0.7	0.4 ± 0.7	0.2398
Bilirubin [mg/dL]	1.2 ± 2.1	1.2 ± 1.9	1.3 ± 2.2	0.5271
AST (GOT) [IU/L]	179 ± 599	190 ± 566	174 ± 616	0.8599
ALT (GPT) [IU/L]	97 ± 195	98 ± 207	97 ± 188	0.4824
**Cardiovascular Values**				
SOFA cardiovascular subscore	1.6 ± 1.0	1.6 ± 1.1	1.6 ± 1.0	0.6821
Patients with vasopressor treatment [%]	81	80	81	0.7154
Vasopressor days/observation days [%]	37 ± 32	36 ± 33	37 ± 31	0.6091
**Central Nervous System**				
SOFA central nervous system	2.1 ± 1.1	2.0 ± 1.1	2.1 ± 1.1	0.1644
Glasgow Coma Scale (GCS)	9.8 ± 3.2	10.0 ± 3.3	9.7 ± 3.2	0.1946
**Renal Values**				
SOFA renal subscore	0.8 ± 1.2	0.9 ± 1.2	0.8 ± 1.2	0.4042
Creatinine [mg/dL]	1.2 ± 0.9	1.3 ± 0.9	1.2 ± 1.0	0.3852
Urine output [mL/d]	2906 ± 1345	2923 ± 1404	2896 ± 1314	0.6595
Urine output [mL/kg/d]	1.5 ± 0.8	1.5 ± 0.8	1.5 ± 0.8	1.0000
Patients with renal replacement therapy [%]	22	23	22	0.9257
Dialysis days/observation days [%]	11 ± 25	11 ± 25	10 ± 24	0.8644

*p*-values calculated using Mann–Whitney U test, Pearson’s chi-square test, or two-sided Fisher’s exact test.

**Table 5:** Multivariate Cox regression analysis (28-day mortality) with regard to TIM-3 rs1036199 genotypes.

Variable	Hazard Ratio	95% CI	*p*-Value
Age	1.0299	1.0163–1.0436	**<0.0001**
Male sex	1.1144	0.7886–1.5746	0.5393
BMI	0.9576	0.9277–0.9884	**0.0073**
SOFA score at sepsis onset	1.0887	1.0333–1.1471	**0.0014**
APACHE II score at sepsis onset	1.0376	1.0043–1.0719	**0.0264**
Use of bronchodilators at sepsis onset	0.9733	0.5734–1.6522	0.9202
TIM-3 rs1036199 AA genotype	0.6714	0.4825–0.9341	**0.0180**

**Table 6:** Multivariate Cox regression analysis (28-day mortality) with regard to TIM-3 rs10515746 genotypes.

Variable	Hazard Ratio	95% CI	*p*-Value
Age	1.0300	1.0165–1.0438	**<0.0001**
Male sex	1.1132	0.7875–1.5736	0.5436
BMI	0.9573	0.9273–0.9882	**0.0071**
SOFA score at sepsis onset	1.0907	1.0354–1.1489	**0.0011**
APACHE II score at sepsis onset	1.0366	1.0035–1.0707	**0.0297**
Use of bronchodilators at sepsis onset	0.9670	0.5699–1.6408	0.9009
TIM-3 rs10515746 CC genotype	0.6981	0.5021–0.9706	**0.0326**

## Supplementary Materials

The following are available online at https://www.mdpi.com/1422-0067/21/21/8318/s1, Supplement 1: Microbiological findings according to TIM-3 rs1036199 genotypes, Supplement 2: Microbiological findings according to TIM-3 rs10515746 genotypes.

## Abbreviations

APACHE	Acute Physiology and Chronic Health Evaluation
BMI	Body mass index
CD4+	CD4-positive T cell
CD8+	CD8-positive cytotoxic T cell
COPD	Chronic obstructive pulmonary disease
CRF	Clinical report form
CRP	C-reactive protein
CTLA-4	Cytotoxic T-lymphocyte associated protein 4
DC	Dendritic cell
Gal-9	Galectin-9
HAVCR2	Hepatitis A virus cellular receptor 2
ICU	Intensive care unit
LD	Linkage disequilibrium
NK	Natural killer cell
NYHA	New York Heart Association
PCR	Polymerase chain reaction
PD-1	Programmed cell death protein 1
SNP	Single nucleotide polymorphism
SOFA	Sequential Organ Failure Assessment
TIM-3	T cell immunoglobulin and mucin domain-containing protein 3
TReg	Regulatory T cells
WHO	World Health Organization
