# Supplementary material for: TIM-3 Genetic Variants Are Associated with Altered Clinical Outcome and Susceptibility to Gram-Positive Infections in Patients with Sepsis
Source: Int J Mol Sci. 2020 Nov 6;21(21):8318. doi: 10.3390/ijms21218318 (PMC7664272; doi:10.3390/ijms21218318)
Supplement: Supplementary file 1 [file ijms-21-08318-s001.pdf]

Supplementary Material

Supplement 1. Microbiological findings according to TIM-3 rs1036199 genotypes

	**All (n=712)**	**AC + CC (n=241)**	**AA (n=471)**	**P-value**
**Type of Infection**				
Gram-positive [%]	76	72	79	**0.0445**
Gram-negative [%]	64	61	66	0.2454
Fungal [%]	52	51	53	0.4999
Viral [%]	11	10	12	0.4401
**Gram-positive Pathogens**				
Staphylococcus epidermidis [%]	32	27	35	**0.0295**
Staphylococcus aureus [%]	22	18	24	0.0792
Enterococcus faecalis [%]	18	15	19	0.1920
Enterococcus faecium [%]	15	17	14	0.2892
**Gram-negative Pathogens**				
Escherichia coli [%]	27	25	28	0.4066
Pseudomonas aeruginosa [%]	12	12	12	0.9590
Klebsiella pneumonia [%]	10	8	11	0.2436
Proteus mirabilis [%]	9	7	10	0.1376
**Fungal Pathogens**				
Candida albicans [%]	30	29	31	0.7596
Candida glabrata [%]	11	10	12	0.4896

Supplement 2. Microbiological findings according to TIM-3 rs10515746 genotypes

	**All (n=712)**	**AA + AC (n=247)**	**CC (n=465)**	**P-value**
**Type of Infection**				
Gram-positive [%]	76	72	78	0.0829
Gram-negative [%]	64	62	66	0.3334
Fungal [%]	52	51	53	0.7055
Viral [%]	11	10	12	0.3495
**Gram-positive Pathogens**				
Staphylococcus epidermidis [%]	32	27	35	**0.0313**
Staphylococcus aureus [%]	22	18	23	0.1072
Enterococcus faecalis [%]	18	16	19	0.2678
Enterococcus faecium[%]	15	17	14	0.2822
**Gram-negative Pathogens**				
Escherichia coli [%]	27	26	28	0.4470
Pseudomonas aeruginosa [%]	12	13	12	0.9101
Klebsiella pneumonia [%]	10	9	10	0.6062
Proteus mirabilis [%]	9	6	10	0.1045
**Fungal Pathogens**				
Candida albicans [%]	30	30	30	0.9200
Candida glabrata [%]	11	11	11	0.7245
